# Association Between Circulating Proprotein Convertase Subtilisin/Kexin Type 9 Concentrations and Cardiovascular Events in Cardiovascular Disease: A Systemic Review and Meta-Analysis

**DOI:** 10.3389/fcvm.2021.758956

**Published:** 2021-11-23

**Authors:** Jiahui Liu, Fangfang Fan, Xingyu Luo, Wenjun Ji, Yaokun Liu, Yan Zhang, Bo Zheng

**Affiliations:** Department of Cardiology, Institute of Cardiovascular Disease, Peking University First Hospital, Beijing, China

**Keywords:** proprotein convertase subtilisin/Kexin type 9, cardiovascular events, secondary prevention, prediction, meta-analysis

## Abstract

**Background:** A large amount of evidence suggests that proprotein convertase subtilisin/Kexin type 9 (PCSK9) inhibitors have clinical benefits in patients with cardiovascular disease (CVD). However, whether PCSK9 concentrations predict future cardiovascular (CV) events remains unclear.

**Methods:** We conducted a meta-analysis to investigate the ability of PCSK9 concentrations to predict future CV events in patients with established CVD. A comprehensive search of electronic databases was conducted in June 2021. We included relative risk (RR) estimates with 95% CI or events of interest.

**Results:** Eleven cohort studies including 8,471 patients with CVD were enrolled. The pooled RR of CV events for the increase in the circulating baseline PCSK9 concentrations by 1 SD showed a positive association in a random-effect model (RR 1.226, 95% CI: 1.055–1.423, *P* = 0.008). Similarly, the risk of the total CV events increased by 52% in the patients in the highest tertile compared with those in the lowest tertile of circulating PCSK9 concentrations (RR 1.523, 95% CI: 1.098–2.112, *P* = 0.012). The association between PCSK9 and CV events was stronger in stable patients with CVD, patients treated with statins, and Asian patients.

**Conclusions:** High PCSK9 concentrations are significantly related to the increased risk of future CV events. These results enrich the knowledge of PCSK9 function and suggest the further possible clinical role of PCSK9 inhibitors.

## Introduction

Cardiovascular disease remains one of the most common causes of morbidity and mortality worldwide. A vital challenge for clinicians managing patients with cardiovascular disease (CVD) is to reduce the risk of recurrent events. Approximately one in five patients is at risk of having a new event during the first year post-acute coronary syndrome (ACS), particularly in older patients (approximately two in five patients) (1, 2). Therefore, identifying specific patients with CVD who have an enhanced risk for future cardiovascular (CV) events is important.

Proprotein convertase subtilisin/Kexin type 9 is a circulating protein that binds to low-density lipoprotein cholesterol (LDL-C) receptors on hepatocytes and targets these receptors for lysosomal degradation. Gain of function proprotein convertase subtilisin/Kexin type 9 (PCSK9) mutations are the third most common genetic cause of autosomal dominant family hypercholesterolemia, and these mutations upregulate LDL-C concentrations and consequently, increase the risk of CVD (3). Recently, therapies directed against PCSK9 have dramatically decreased LDL-C concentrations and reduced CV events under statin treatment (4). Although PCSK9 inhibitors are established as novel targets that lower lipids and result in clinical improvement (5, 6), the value of PCSK9 concentrations as a predictor for CV events is unclear. Although PCSK9 inhibitors (e.g., alirocumab and evolocumab) are recommended for lipid-lowering to attain the target LDL-C goal (7), knowledge regarding the circulating PCSK9 concentrations for CV risk stratification is lacking.

Previous studies on the association between PCSK9 and CV events are conflicting (8, 9), partly because of relatively small sample sizes. Moreover, meta-analyses on the relationship between PCSK9 and CV events were focused on the general population (10, 11). Therefore, we conducted this meta-analysis to provide more comprehensive evidence on the predictive value of PCSK9 in patients with established CVD.

## Materials and Methods

### Search Strategy and Study Selection

This meta-analysis was reported according to the Preferred Reporting Items for Systematic reviews and Meta-Analyses (PRISMA) statement (12) and was registered at the International Prospective Register of Systematic Reviews (number CRD42021259074). The following databases were searched: Cochrane Central Register of Controlled Trials, Medical Literature Analysis and Retrieval System Online (MEDLINE), and Excerpta Medica dataBASE (EMBASE) on June 10, 2021. The medical subject headings and keywords are shown in the Supplementary Material (see search strategy).

The main inclusion criteria were as follows: (1) serum PCSK9 concentrations were detected in patients with CVD; (2) CV outcomes of interest were reported; (3) and full-length publications. The trials performed in patients with familial hypercholesterolemia or the general population without established CVD were excluded. There were no restrictions on language. The references of the relevant articles were also manually checked to avoid missing articles. Two investigators (Liu JH, Luo XY) who were not involved in any of the selected trials independently searched the databases, abstracted the prespecified data, and assessed the potential risks of bias using the Newcastle–Ottawa scale. Any discrepancies were resolved by consensus after discussions with a third investigator (Zheng B). The following data were abstracted: the name of the author and year published, size and type of the cohort, characteristics of the patients and follow-up duration, PCSK9 assay methods and collection time, hazard ratios (HRs) or relative risks (RRs) with 95% CIs, and adjustment for potential confounders.

We used the Newcastle-Ottawa scale to evaluate each included study regarding the following three criteria: (1) selection of patients; (2) comparability of the patients; (3) ascertainment of the exposure. Studies that scored ≥7 (out of a maximum of nine points) were considered to be high quality.

### Outcome

The primary endpoint was composite CV events, including all-cause death, myocardial infarction (MI), revascularizations (coronary artery bypass graft surgery, coronary percutaneous interventions), and stroke (detailed endpoints across studies are listed in the Supplementary Table 1).

### Data Synthesis and Statistical Analysis

The risk estimates of the association between PCSK9 concentrations and CV events in this meta-analysis are reported as RRs with 95% CIs. Hazard ratios or RRs with 95% CIs were reported in the included studies. We treated HRs as RRs, which have been commonly used in previous studies (13). Multi-adjusted RRs were pooled across the included studies in this meta-analysis (detailed adjustments across studies are listed in the Supplementary Table 2). Both continuous (per one unit or SD increase) and categorical (tertiles or quartiles) variables of PCSK9 were reported in the included studies. To provide a more meaningful effect size of the results, we transformed the RR of each study to standard risk estimates for a 1-SD increase in PCSK9 concentrations We also compared the highest tertile with the lowest tertile for the distribution of PCSK9 concentrations using methods as described previously (10, 11, 14). The conversion assumed that PCSK9 was log-normally distributed and had a log-linear association with the outcome. The RRs reported as continuous values were converted as 2.18 times the log RR for a 1-SD difference.

The statistical heterogeneity was assessed using the Cochran Q test (*P* < 0.10 was considered to be statistically significant) and the *I*^2^ statistic (low heterogeneity, *I*^2^ < 50%; moderate heterogeneity, *I*^2^ ≥ 50% and <75%; high heterogeneity, *I*^2^ ≥ 75%) (15). The pooled RRs were calculated using the random-effects model. The clinical type, statin use, PCSK9 measurement, sample source, sample size, and race of the patients were potential clinical causes of heterogeneity. Therefore, we created subgroups to assess the certainty of the evidence. We divided the studies into the ACS group (if > 80% of patients had ACS) and the stable CVD group. Based on statin use before admission, we divided the studies into the statin use group and the non-statin group.

Sensitivity analyses were performed to evaluate the robustness of the association between baseline PCSK9 concentrations and outcomes. We assessed the effect of each individual study on the overall risk estimate by excluding one study at a time (16).

The potential publication bias was examined by constructing a funnel plot in which the SE of the log RR was plotted against the RR. The asymmetry of the plot was estimated visually and quantitatively using Begg's rank correlation test and Egger's linear regression test (17, 18).

The analyses were conducted using STATA version 14.0 (Stata Corp LP, College Station, Texas, United States) wherein *P* < 0.05 (two-tailed) was considered statistically significant.

## Results

### Study Selection and Patient Population

**Figure 1:**
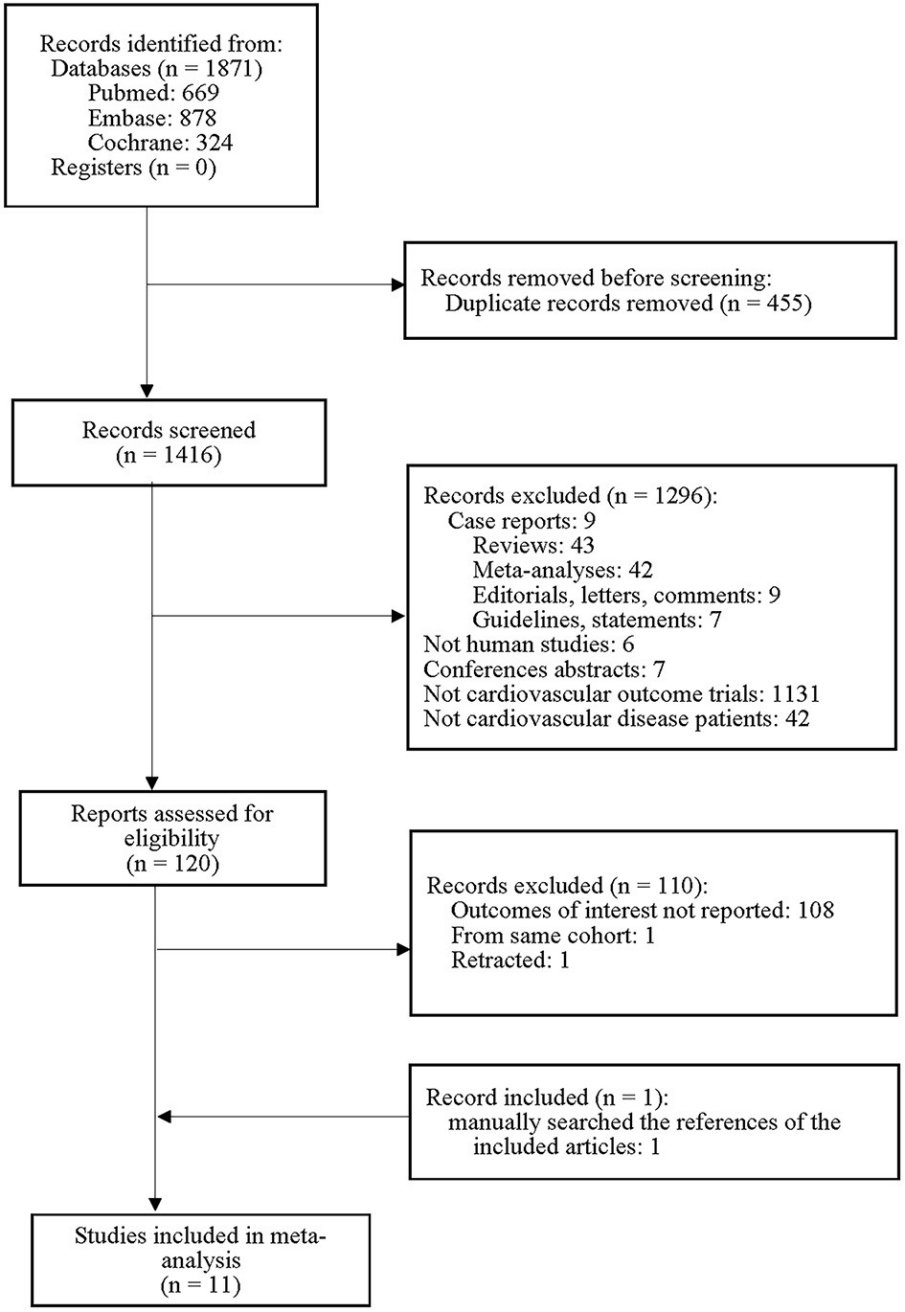
Flowchart for selection of the studies.

The PRISMA flow diagram of the meta-analysis is shown in Figure 1. Of the 1,871 studies initially identified, 1,296 were excluded on the basis of the title and abstract content. A total of 110 studies did not meet the explicit inclusion criteria. One additional trial was identified by reviewing the references of previous studies. Finally, 11 studies were included in this meta-analysis.

Table 1 shows the characteristics of the included studies (8, 9, 19–24, 26–28). Of the 11 studies included, only 1 (22) was a retrospective cohort study and the others were prospective cohort studies, with a publication period from 2014 to 2020. Four trials (20, 22, 27, 28) reported RRs according to continuous concentrations of PCSK9, three studies (19, 24, 26) reported RRs according to categorical levels, and four studies (8, 21, 23, 28) reported RRs according to both continuous and categorical levels. We included 8,471 patients, and the sample size of each study ranged from 249 to 2,030. The longest follow-up was 5.35 years (20), and the mean weighted follow-up was 1.42 years. The baseline serum PCSK9 concentrations ranged from 52.1 to 607 ng/ml (weighted mean, 283.8 ng/ml). None of the patients included in this meta-analysis were treated with drugs targeting PCSK9.

**Table 1:** Characteristics of the included studies.

**Study**	**Study design**	**Population**	**Sample size**	**Country**	**Age (years, mean or median)**	**Male (%)**	**Reperfusion (%)**	**Follow-up (years, mean or median)**	**PCSK9 concentration (mean ± SD or median and IQR, ng/ml)**	**PCSK9 measurement method**	**Blood sampling**	**Events**	**Statin use (%)**
Choi et al. (19)	Prospective cohort	CAD underwent PCI (19.2% had stable AP, 29.9% had UA, 32.0% had NSTEMI, 16.0% had STEMI, 1.87% had silent myocardial ischemia)	749	Korea	65.9	66.5	100	2.34	302.82 (234.30–366.91)	Human PCSK9 Quantikine Kit (R&D Systems, Inc., Minneapolis, Minnesota)	Upon arrival at the catheterization laboratory before PCI	MACE occurred in 38 patients (5.1%), and 50 patients (6.7%) died	31.4
Franco-Peláez et al. (20)	Prospective cohort	AMI undergoing CAG (46.7% had STEMI, 53.3% had NSTEMI)	270	Spain	65.0	66.7	70.0	5.35	52.1 (42.4–64.3)	In duplicate by ELISA method with anti-PCSK9 specific antibodies (ELLA kit, R&D Systems, Minneapolis, MN, USA)	6–12 months after AMI	40 had a first AAE (NSTEACS, STEMI, stroke, or TIA), 25 ACS, 15 stroke/TIA	93.0
Peng et al. (21)	Prospective cohort	First diagnosed stable CAD by CAG or CTA	1,225	China	57.8	68.0	/	3.3	234.52 (194.79–276.13)	Quantitative sandwich enzyme immunoassay (Quantikine ELISA, R&D Systems Europe Ltd)	Overnight fasting immediately after their admission	103 MACEs (three had non-fatal MI, 41 underwent coronary revascularization, eight had IS, six died and 45 suffered hospitalization because of UA)	0
Zhang et al. (22)	Retrospective cohort	AMI underwent CAG (61.6% had STEMI, 38.4% had NSTEMI)	281	China	Male: 58.53, Female: 68.64	78.3	/	1	283.8 (227.7–393.3)	Quantitative sandwich enzyme immunoassay ELISA (catalog number Circulex CY-8079; CycLex Co., Ltd., Japan)	Prior to CAG	18 episodes of MACE occurred (one had recurrent AMI, four had TVR, one had stroke, 12 had cardiac death)	18.9
Cao et al. (23)	Prospective cohort	FH patients with angiography- proven CAD who had experienced a first CVE (MI, stroke, UA, PCI, CABG, peripheral arterial revascularization)	249	China	50.23	56.2	/	3.58	303.66 (240.25–396.34)	Quantitative sandwich enzyme immunoassay (Quantikine ELISA, R & D Systems Europe Ltd)	Fasting	29 had recurrent CVEs, three had MI, four had stroke, 12 had revascularization, 10 had cardiac death	88
Gao et al. (24)	Prospective cohort	AMI within 24 h of symptom onset	1,646	China	61	74.8	47.5	1	271.3 during the acute phase, 380.9 at 1 month, and 355.1 at 1 year	Colorimetric enzyme-linked immunosorbent assay (Human PCSK9 Immunoassay, DPC900, R&D Systems)	Within 24 h of symptom onset (mean 6 h), prior to the initiation of statin therapy and CAG	37 experienced cardiac death, 27 non-fatal AMI, 82 coronary revascularization, and 10 IS	0
Cheng et al. (25)	Prospective cohort	CAD underwent CAG or PCI (28.5% had STEMI, 26% had NSTEACS, 45.5% had stable CAD)	581	Europe	61.5	75.5	88	1	270 (217–336)	Enzyme linked immunosorbent assay (Human PCSK9 Quantikine ELISA, R&D Systems Inc., Minneapolis, MN, USA)	Prior to CAG	28 patients died or had an ACS	62.3
Navarese et al. (26)	Prospective cohort	ACS (60.06% had STEMI, 33.03% had NSTEMI, 6.91% had UA)	333	Austria	57.0	79.9	84.7	1	/	Quantitative sandwich enzyme immunoassay (CircuLex™ Human PCSK9 ELISA kit, Japan)	One day after the CAG	13 (22.03%) in the upper PCSK9 tertile experienced a clinical MACE (CV death, MI, UA, stent thrombosis, repeat revascularization and IS), 2 (3.39%) in the lower PCSK9 tertile	91.9
Li et al. (27)	Prospective cohort	Non-treated stable CAD underwent CAG	603	China	57.88	72.0	52.4	1.42	230.11 (190.45–277.83)	Quantitative sandwich enzyme immunoassay (Quantikine ELISA, R & D Systems Europe Ltd, Minneapolis, USA)	Fasting	72 had at least one MACE (four cardiac deaths, four non-fatal strokes, six MIs, 28 revascularizations, and 30 UAs)	0
Gencer et al. (9)	Prospective cohort	ACS underwent CAG (52.9% had STEMI, 43.0% had NSTEMI, 4.1% had UA)	2,030	Swiss	63.6	78.9	/	1	323 ± 134	Colorimetric enzyme-linked immunosorbent assay from R&D Systems (Minneapolis, MN, USA)	At CAG	30 ACS patients died in the lowest tertile (4.4%), 26 in the middle tertile (3.8%), and 34 in the highest tertile	30
Werner et al. (8)	Prospective cohort	Stable CAD documented by CAG	504	Germany	68	83	/	4	Male: 607 (498–764); Female: 532 (427–652)	Enzyme-linked immunosorbent assay using the CircuLex Human PCSK ELISA Kit (CY-8079, CycLex, Japan)	Fasting	362 primary outcomes occurred (CV death and CV hospitalization for ACS)	95

*CAD, coronary artery disease; PCI, percutaneous coronary intervention; AP, angina pectoris; UA, unstable angina; NSTEMI, non-ST-segment elevation myocardial infarction; STEMI, ST-segment elevation myocardial infarction; MACE, major adverse cardiovascular events; AMI, acute myocardial infarction; CAG, coronary angiography; AAE, acute atherothrombotic events; NSTEACS, non-ST-segment elevation acute coronary syndrome; TIA, transitory ischemic attack; CTA, computed tomography angiography; IS, ischemic stroke; TVR, target vessel revascularization; FH, familial hypercholesterolemia; CVE, cardiovascular event; CABG, coronary artery bypass graft*.

### Association Between PCSK9 and CV Events

#### Circulating PCSK9 Concentrations as a Continuous Variable

**Figure 2:**
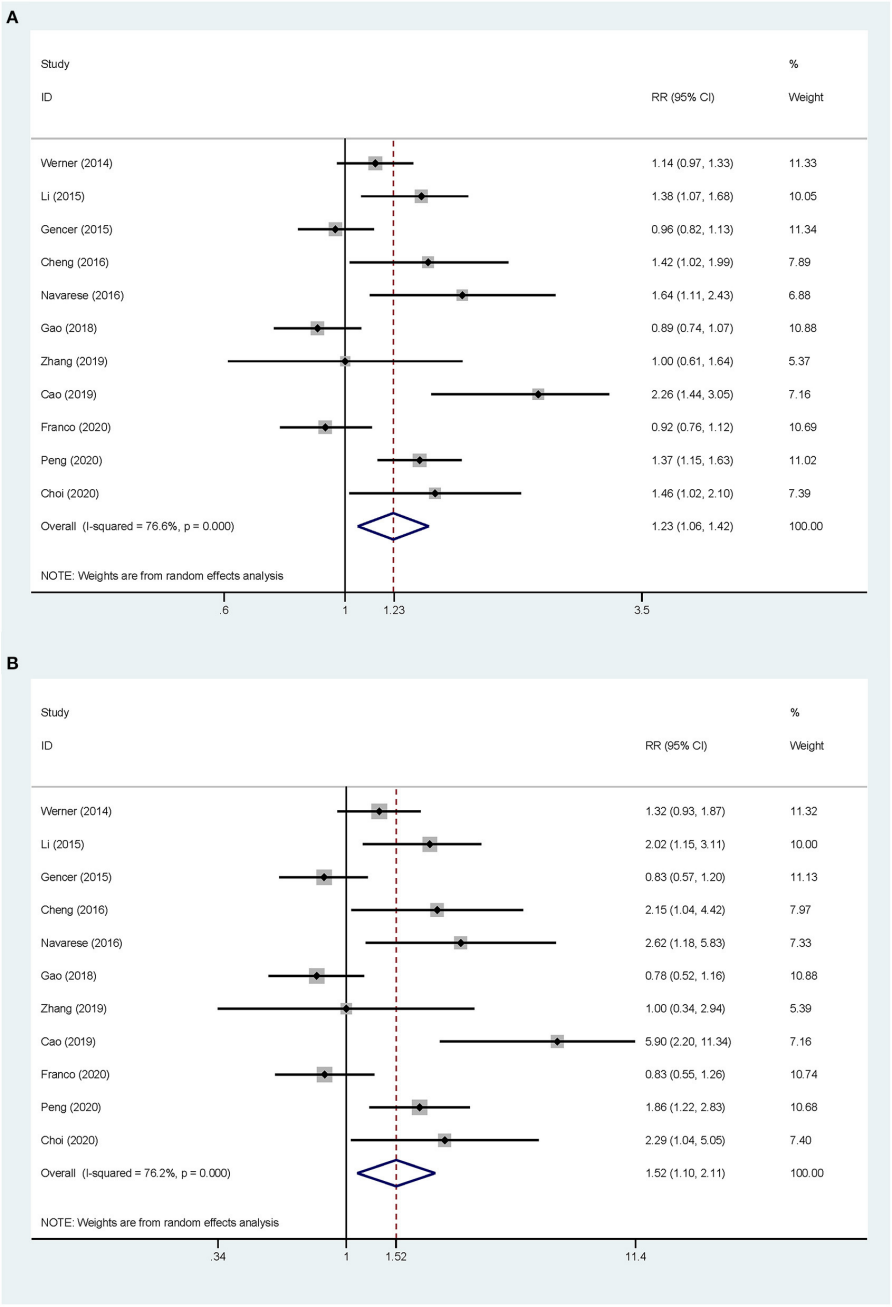
Association between the circulating proprotein convertase subtilisin/Kexin type 9 (PCSK9) concentrations and the risk of cardiovascular (CV) events. **(A)** The relative risks (RR) of CV events per 1-SD increase in the baseline PCSK9 concentrations. **(B)** Top vs. bottom tertile of the baseline PCSK9 concentrations.

**Figure 3:**
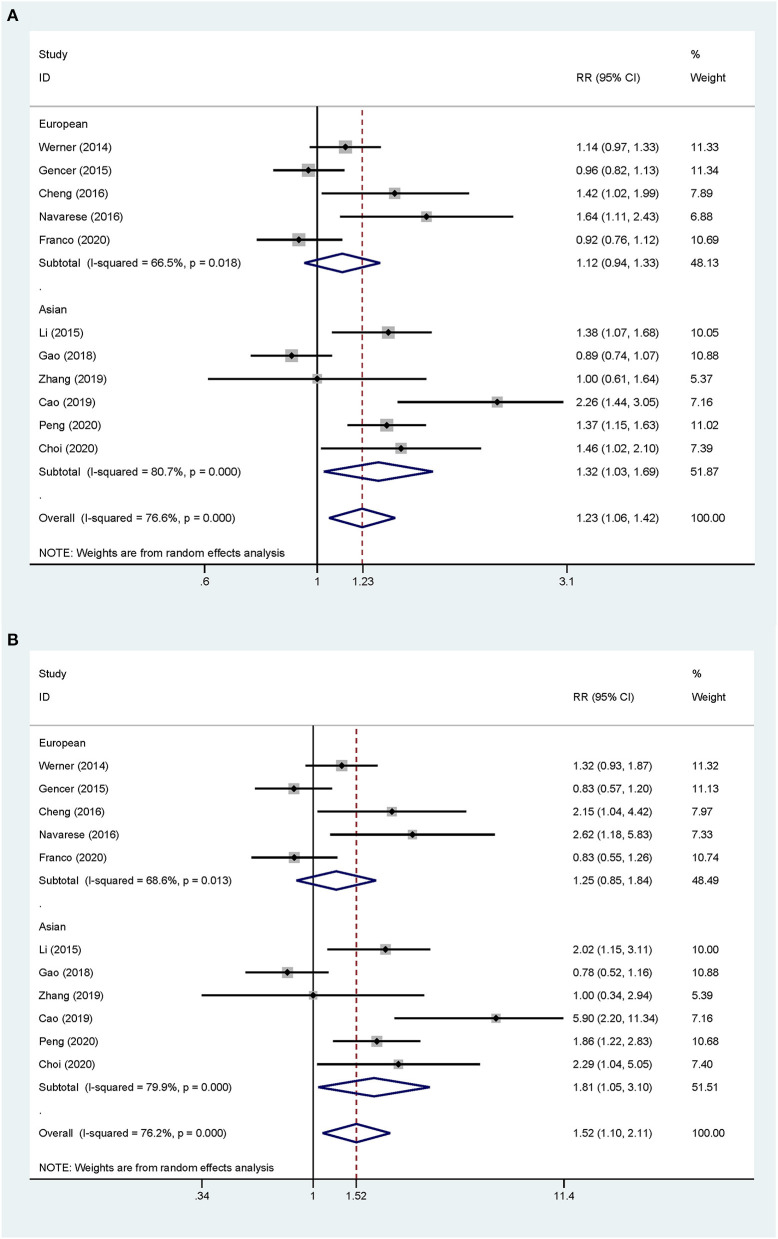
Subgroup analyses for circulating PCSK9 concentrations and the risk of CV events. **(A)** The RR of CV events per 1-SD increase in the baseline PCSK9 concentrations. **(B)** Top vs. bottom tertile of the baseline PCSK9 concentrations.

**Figure 4:**
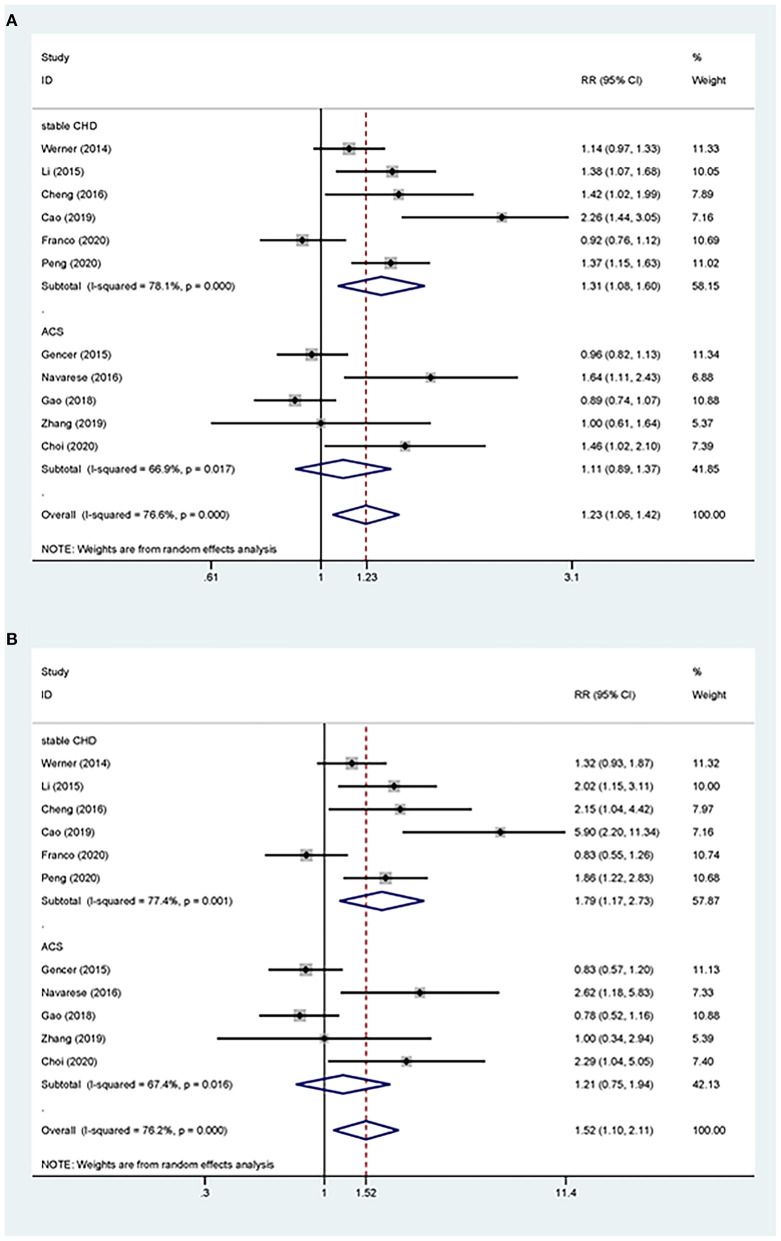
Subgroup analyses for circulating PCSK9 concentrations and the risk of CV events. **(A)** The RR of CV events per 1-SD increase in the baseline PCSK9 concentrations. **(B)** Top vs. bottom tertile of the baseline PCSK9 concentrations.

**Figure 5:**
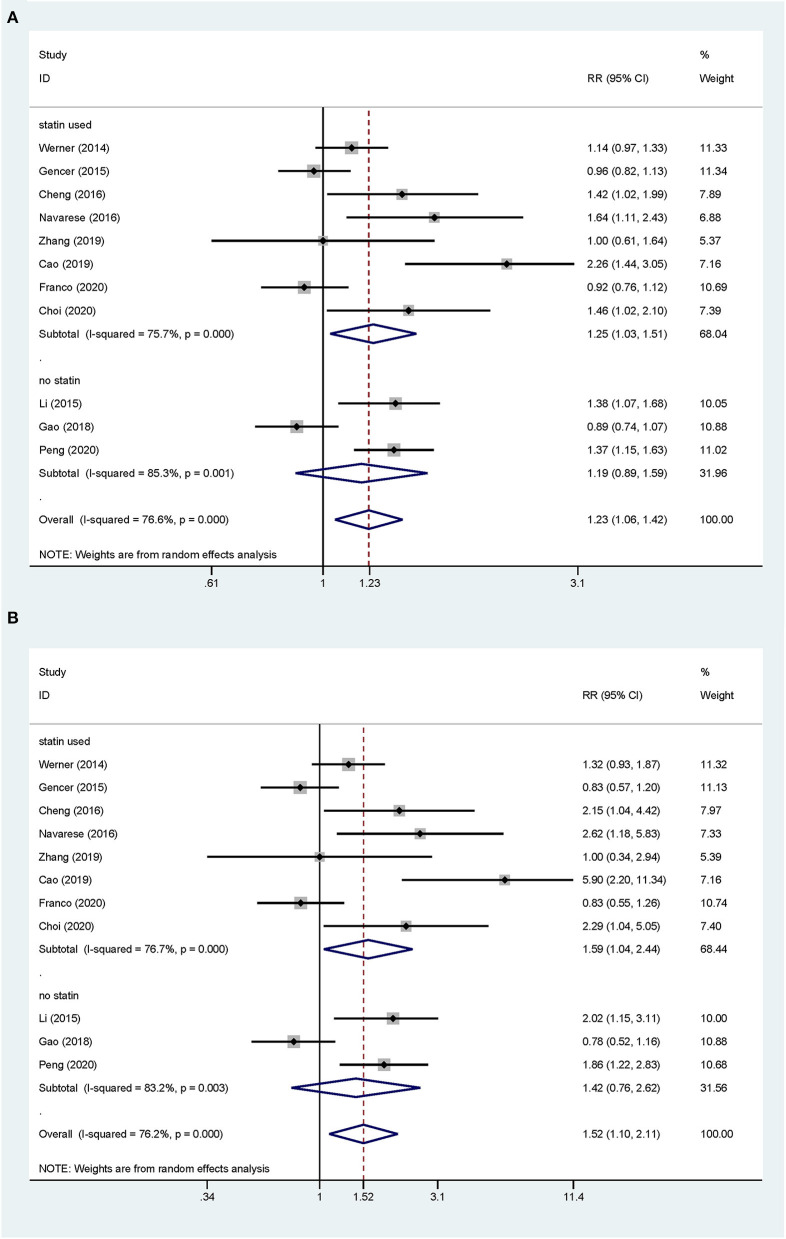
Subgroup analyses for circulating PCSK9 concentrations and the risk of CV events. **(A)** The RR of CV events per 1-SD increase in the baseline PCSK9 concentrations. **(B)** Top vs. bottom tertile of the baseline PCSK9 concentrations.

Overall, the pooled RR of CV events for the increase in the circulating baseline PCSK9 concentrations by 1 SD showed a positive association in the random-effect model (RR 1.226, 95% CI: 1.055–1.423, *P* = 0.008) (Figure 2A). We further conducted preset subgroup analyses because of the significant heterogeneity between the included studies. This positive association was much stronger in the studies conducted in Asian patients (RR 1.319, 95% CI: 1.030–1.689, *P* = 0.028) compared with European patients (RR 1.120, 95% CI: 0.943–1.329, *P* = 0.195) (Figure 3A). Notably, the heterogeneity was also significantly reduced, which indicated that race may have contributed to the source of heterogeneity. A positive association between PCSK9 and CV events was only observed in patients with stable CVD (RR 1.313, 95% CI: 1.080–1.597, *P* = 0.006) but not in patients with ACS (RR 1.106, 95% CI: 0.892–1.373, *P* = 0.359) (Figure 4A). The PCSK9 concentrations significantly predicted the CV events in the statin use group but not in the non-statin group (RR 1.250, 95% CI: 1.033–1.513, *P* = 0.022; RR 1.186, 95% CI: 0.885–1.589, *P* = 0.252 respectively) (Figure 5A). No significant associations were observed between the risk of CV events and Cyclex kit (Japan) PCSK9 assay measurement (see Supplementary Figure 1A) and the small sample size (<500 patients included) (see Supplementary Figure 2A). Additionally, heterogeneity was reduced in the subgroup analysis of the Cyclex kit (Japan) PCSK9 assay measurement with a large sample size (>500 patients), which suggested that these factors contributed to heterogeneity.

In a sensitivity analysis, the exclusion of any single study did not substantially alter the combined risk estimate (see Supplementary Figure 3), which suggested that the overall association was robust.

#### Circulating Concentrations of PCSK9 as an Ordinal Variable

The total CV event risk was increased by 52% in patients who were in the highest tertile compared with those in the lowest tertile of circulating PCSK9 concentrations (RR 1.523, 95% CI: 1.098–2.112, *P* = 0.012) (Figure 2B). Significant heterogeneity was observed across the studies (*I*^2^ = 76.2%, *P* < 0.001), and pooled effects were derived under the random-effect model meta-analysis. The subgroup and sensitivity analyses for the category PCSK9 showed the same results as those in the analysis of the increase in the circulating baseline PCSK9 concentrations by 1 SD (Figures 2–5; Supplementary Figures 1, 2).

In a further meta-regression analysis, race, sex, age at study recruitment, follow-up years, sample size, presence of ACS, sample source, statin use, assays for PCSK9, and PCSK9 concentrations were not predictors of the magnitude of the RR for total CV events (*P* = 0.436, 0.136, 0.781, 0.685, 0.156, 0.357, 0.649, 0.770, 0.987, and 0.763, respectively).

### Risk of Bias

The assessment of the risk of bias for each included study using the Newcastle - Ottawa Quality Assessment Scale (NOS) is shown in the Supplementary Table 3. Six studies scored a maximum of nine points and the other five scored eight points.

Although the funnel plot for the association between circulating PCSK9 concentrations and CV events was asymmetrical at the bottom left of the plot (see Supplementary Figure 4), the regression tests for funnel plot asymmetry did not show publication bias (Begg's test, *P* = 0.213; Egger's test, *P* = 0.058).

## Discussion

In this meta-analysis, 11 cohort studies including 8,471 patients with CVD were enrolled. To the best of our knowledge, this is the first meta-analysis to investigate the predictive effect of circulating PCSK9 concentrations on the secondary prevention of CVD. Our study showed that patients with established CVD and high PCSK9 concentrations had a 52% higher risk of future total CV events than those with low PCSK9 concentrations. An increase in the PCSK9 concentrations by 1 SD was associated with a 23% increased risk of future total CV events. Moreover, this predictive effect was stronger in patients who received statin therapy, stable CVD, and Asian patients.

Besides the lipid-lowering effect of PCSK9, it plays a major role in the inflammatory reaction, platelet aggregation, and endothelial apoptosis contributing to atherosclerosis (29–31). Among the top 10 genetic variants associated with elevated LDL-C concentrations, a PCSK9 variant had the weakest association with elevated LDL-C concentrations but had the third strongest effect on the risk of MI (32). Experimental studies have shown that PCSK9 is expressed in human atherosclerotic plaques (33). Clinical trials have shown that higher serum PCSK9 concentrations are linearly associated with a higher necrotic core fraction in coronary atherosclerosis, independent of serum LDL-C concentrations and statin use (25). Previous studies have suggested that PCSK9 concentrations are higher in patients with CVD and is a potential biomarker of the severity of CVD (34–37). Patients with ACS and higher PCSK9 concentrations are less likely to reach the recommended LDL-C targets during 1 year of follow-up (9).

While there is strong experimental and clinical evidence that PCSK9 has a direct effect on the atherosclerosis pathway, the clinical utility of PCSK9 regarding risk stratification remains unclear. Previous meta-analyses have shown conflicting findings. Vlachopoulos et al. (11) found that high PCSK9 concentrations were associated with an increased risk of CV events in the general population, but not in the high-risk population. However, similar significant positive associations were observed in low- and high-risk CV subgroups by Qiu et al. (38) and Zhou et al. (10). The small number of included high CV risk studies and the relatively high heterogeneity of the high CV risk population may explain these contrasting findings. Recently, there are five relative clinical trials published, and we only studied patients with CVD. The positive association between PCSK9 concentrations and total CV events remained in patients with stable CVD, but not in patients with ACS. Several factors are proposed to explain this result. Previous studies have shown that PCSK9 concentrations are transiently upregulated in the acute period of MI (39, 40), but PCSK9 concentrations drop back to a plateau in the long term (40). Hepatic PCSK9 expression is enhanced in MI and inflammation. Therefore, blood sampling in the acute ACS period could bias the prognostic value of PCSK9 concentrations. An inflammatory burden in ACS might lead to a prognostic bias because PCSK9 participates in the inflammatory process. Therefore, PCSK9 concentrations in the acute clinical setting are less likely to predict future events.

Another common factor that could bias the prognostic value of PCSK9 concentrations is the statin status. Many studies have shown that circulating PCSK9 concentrations rapidly rise after initiating statin therapy (9, 37), and there is a sustained increase throughout statin use. In one study, 80 mg of atorvastatin led to a rapid 47% increase in the serum PCSK9 concentrations, and this significant increase was sustained throughout 16 weeks of dosing (41). An increase in plasma PCSK9 concentrations results in a partial attenuation of the effects of statin on LDL receptor expression, which appears to be responsible for statin resistance (42, 43). Our subgroup analysis supports this mechanism. Proprotein convertase subtilisin/Kexin type 9 concentrations showed a positive relationship with CV events in the statin use subgroup, whereas this relationship was not present in the non-statin subgroup. This finding suggests the potential enrichment of the current indication for PCSK9 inhibitors.

Our study showed a strong positive association between PCSK9 concentrations and future CV events in Asian patients. The frequency of PCSK9 variations and concentrations vary among different ethnic groups (44, 45). The PCSK9 R46L and A443T variants are associated with lower LDL-C concentrations. The PCSK9 R46L variant is more frequent in Caucasian populations than in African Americans, while the A443T variant is rarer (minor allele frequency in Caucasian populations is 1.6 vs. 0.28% in African Americans and 0.048 vs. 9.4%, respectively). The minor allele frequencies of the PCSK9 c.61_63insCTG variant (denoted L10Ins) are comparable between Caucasians and Japanese (11 vs. 15%), but Caucasians are predisposed to having low LDL-C concentrations (46–48). According to recent meta-analyses, a reduction in the LDL-C concentrations with PCSK9 monoclonal antibodies is not affected by race (5, 49). Notably, the individuals who were enrolled in randomized, controlled trials of PCSK9 inhibitors were mainly from Western countries. In the Further CV Outcomes Research with PCSK9 Inhibition In subjects With Elevated Risk (FOURIER) and Evaluation of Cardiovascular Outcomes After an ACS During Treatment With Alirocumab (ODYSSEY) trials, 80% of the patients were White individuals and only 13% were Asian. Therefore, the efficacy of PCSK9 inhibitors between different races should be viewed with caution. Racial differences in response to statins are well-known. The SLCO1B1^*^15 haplotype results in higher serum simvastatin concentrations and a higher rate of myopathy, and it occurs at a frequency of 17% in Japanese vs. 1% in Black individuals (50). Our study suggested that the effects of PCSK9 vary in different races. In particular, more studies of these effects in Asian patients are required.

Currently, there is no gold standard measurement method for PCSK9. Sample sources from serum and plasma and the ELISA technique are used for PCSK9 measurement. The variance in the measurement methods of PCSK9 may explain the diverse PCSK9 concentrations across studies. Although we performed a sensitivity analysis, the potential methodological problems from the original studies cannot be eliminated. There are two forms of PCSK9 in the plasma, namely, furin-cleaved and uncleaved forms; furin-cleaved PCSK9 is probably biologically inactive. The most widely used and commercially available technique of ELISA cannot distinguish these two forms. Our study highlights the importance of establishing a standard measurement method for PCSK9 that assists popular clinical practices.

Although PCSK9 might be a surrogate biomarker of future CV events in patients with CVD, there are insufficient data for a definite conclusion. Our study enriches the knowledge of PCSK9 function and highlights the requirement for more studies to investigate PCSK9 while taking race and an accurate method into account.

## Limitations

There are several limitations to this meta-analysis. First, the pooled data were extracted from published articles rather than data for the individual patients being used, thus we could not make more specific stratification and we are unable to detect a single outcome in this meta-analysis. Second, the adjustments for models and CV event definitions differed among the included studies. Therefore, we conducted the random-effects model and subgroup analysis to minimize the potential heterogeneity. Finally, there was a lack of baseline lipid profile and C-reactive protein concentration data, and the meta-regression techniques used were limited in the present analysis. Therefore, the effect of these factors on the predictive ability of PCSK9 cannot be totally evaluated.

## Conclusion

Proprotein convertase subtilisin/Kexin type 9 concentrations are positively significantly associated with future CV events in patients with established CVD. This finding supports the clinical benefits of PCSK9 inhibitors and suggests that PCSK9 concentrations are important for improving risk stratification for medical decisions.

## Data Availability Statement

The raw data supporting the conclusions of this article will be made available by the authors, without undue reservation.

## Author Contributions

JL and BZ designed the study. JL and XL collected and extracted the data. WJ and YL contributed to the literature search. JL and FF performed the statistical analysis. JL drafted the manuscript. BZ and YZ performed the major revisions of the manuscript. All authors read and approved the final manuscript.

## Conflict of Interest

The authors declare that the research was conducted in the absence of any commercial or financial relationships that could be construed as a potential conflict of interest.

## Publisher's Note

All claims expressed in this article are solely those of the authors and do not necessarily represent those of their affiliated organizations, or those of the publisher, the editors and the reviewers. Any product that may be evaluated in this article, or claim that may be made by its manufacturer, is not guaranteed or endorsed by the publisher.
