# Supplementary material for: Association Between Circulating Proprotein Convertase Subtilisin/Kexin Type 9 Concentrations and Cardiovascular Events in Cardiovascular Disease: A Systemic Review and Meta-Analysis
Source: Front Cardiovasc Med. 2021 Nov 23;8:758956. doi: 10.3389/fcvm.2021.758956 (PMC8650021; doi:10.3389/fcvm.2021.758956)
Supplement: Supplementary file 1 [file Table_1.DOCX]

**Search strategies**

- 1. Search strategy for Pubmed

#1 "Proprotein Convertase 9"[Mesh]

#2 (((((((((((((((((Convertase 9, Proprotein[Text Word]) OR (Neural Apoptosis-Regulated[Text Word]) OR (Convertase 1[Text Word]) OR (Neural Apoptosis Regulated Convertase 1[Text Word]) OR (neural apoptosis regulated convertase-1[Text Word]) OR (NARC-1 Protein

NARC 1 Protein[Text Word]) OR (NARC-1[Text Word]) OR (NARC1[Text Word]) OR (Proprotein Convertase, Subtilisin-Kexin Type 9[Text Word]) OR (Proprotein Convertase, Subtilisin Kexin Type 9[Text Word]) OR (PCSK9 protein[Text Word]) OR (protein PCSK9[Text Word]) OR (proprotein convertase subtilisin/kexin type 9[Text Word]) OR (Pro-protein convertase subtilisin-kexin type 9[Text Word]) OR (proprotein convertase subtilisin kexin 9[Text Word]) OR (PCSK9[Text Word])

#3 #1 OR #2

#4 "Cardiovascular Diseases"[Mesh]

#5 ((((((((((((((((((((((((((((((((("cardiovascular disease"[Text Word]) OR "CVD"[Text Word]) OR "cardiovascular risk"[Text Word]) OR "cardiovascular events"[Text Word]) OR "acute coronary syndromes"[Text Word]) OR "ACS"[Text Word]) OR "coronary artery disease"[Text Word]) OR "CAD"[Text Word]) OR "coronary heart disease"[Text Word]) OR "CHD"[Text Word]) OR "ischemic heart disease"[Text Word]) OR "ischemic heart disease"[Text Word]) OR "myocardial infarction"[Text Word]) OR "MI"[Text Word]) OR "myocardial ischemia"[Text Word]) OR "myocardial ischaemia"[Text Word]) OR "coronary stenosis"[Text Word]) OR "coronary restenosis"[Text Word]) OR "heart failure"[Text Word]) OR "cerebrovascular disease"[Text Word]) OR "cerebrovascular disorder"[Text Word]) OR "stroke"[Text Word]) OR "cerebral infarction"[Text Word]) OR "intracranial arteriosclerosis"[Text Word]) OR "brain ischemia"[Text Word]) OR "transient ischemic attacks"[Text Word]) OR "TIA"[Text Word]) OR "intracranial hemorrhage"[Text Word]) OR "hemorrhagic stroke"[Text Word]) OR "death" [Text Word]) OR "all-cause death"[Text Word]) OR mortality[Text Word]) OR "all-cause mortality"[Text Word]) OR "cardiovascular mortality"[Text Word]

#6 #4 OR #5

#7 “epidemiologic studies”[MeSH Terms] OR “cohort studies”[MeSH Terms]

#8 ((((((epidemiologic[Text Word]) OR cohort[Text Word]) OR longitudinal[Text Word]) OR “follow up”[Text Word]) OR observational[Text Word]) OR prospective[Text Word])))

#9 #7 OR #8

#10 #3 AND #6 AND #9

- 1. Search strategy for Embase

#1 'proprotein convertase 9'/exp

#2 'proprotein convertase 9':ab,ti OR 'convertase 9, proprotein':ab,ti OR 'proprotein convertase, subtilisin-kexin type 9':ab,ti OR 'proprotein convertase, subtilisin kexin type 9':ab,ti OR 'proprotein convertase subtilisin/kexin type 9':ab,ti OR 'pro-protein convertase subtilisin-kexin type 9':ab,ti OR 'proprotein convertase subtilisin kexin 9':ab,ti OR 'pcsk9':ab,ti OR 'pcsk9 protein':ab,ti OR 'protein pcsk9':ab,ti OR 'neural apoptosis-regulated convertase 1':ab,ti OR 'neural apoptosis regulated convertase 1':ab,ti OR 'narc-1 protein':ab,ti OR 'narc 1 protein':ab,ti OR 'narc-1':ab,ti

#3 #1 OR #2

#4 'cardiovascular disease'/exp

#5 'cardiovascular disease':ab,ti OR 'cvd':ab,ti OR 'cardiovascular risk':ab,ti OR 'cardiovascular events':ab,ti OR 'acute coronary syndromes':ab,ti OR 'acs':ab,ti OR 'coronary artery disease':ab,ti OR 'cad':ab,ti OR 'coronary heart disease':ab,ti OR 'chd':ab,ti OR 'ischemic heart disease':ab,ti OR 'ischaemic heart disease':ab,ti OR 'myocardial infarction':ab,ti OR 'mi':ab,ti OR 'myocardial ischemia':ab,ti OR 'myocardial ischaemia':ab,ti OR 'coronary stenosis':ab,ti OR 'coronary restenosis':ab,ti OR 'heart failure':ab,ti OR 'cerebrovascular disease':ab,ti OR 'cerebrovascular disorder':ab,ti OR 'stroke':ab,ti OR 'cerebral infarction':ab,ti OR 'intracranial arteriosclerosis':ab,ti OR 'brain ischemia':ab,ti OR 'transient ischemic attacks':ab,ti OR 'tia':ab,ti OR 'intracranial hemorrhage':ab,ti OR 'hemorrhagic stroke':ab,ti OR 'death':ab,ti OR 'all-cause death':ab,ti OR 'mortality':ab,ti OR 'all-cause mortality':ab,ti OR 'cardiovascular mortality':ab,ti

#6 #4 OR #5

#7 'prospective study'/exp

#8 'cohort analysis'/exp

#9 'cohort analysis':ab,ti OR 'cohort study':ab,ti OR 'longitudinal':ab,ti OR 'follow up':ab,ti OR 'observational':ab,ti OR 'prospective':ab,ti OR 'prospective study':ab,ti

#10 #7 OR #8 OR #9

#11 #3 AND #6 AND #10

1.3 Search strategy for Cochrane

#1 MeSH descriptor: [Proprotein Convertase 9] explode all trees

#2 (proprotein convertase 9):ab,ti,kw OR (convertase 9, proprotein):ab,ti,kw OR (proprotein convertase, subtilisin-kexin type 9):ab,ti,kw OR (proprotein convertase, subtilisin kexin type 9):ab,ti,kw OR (pro-protein convertase subtilisin-kexin type 9):ab,ti,kw OR (proprotein convertase subtilisin kexin 9):ab,ti,kw OR (PCSK9):ab,ti,kw OR (PCSK9 protein):ab,ti,kw OR (protein PCSK9):ab,ti,kw OR (neural apoptosis-regulated convertase 1):ab,ti,kw OR (neural apoptosis regulated convertase 1):ab,ti,kw OR (NARC-1 protein):ab,ti,kw OR (NARC 1 protein):ab,ti,kw OR (NARC-1):ab,ti,kw

#3 #1 or #2

#4 MeSH descriptor: [Cardiovascular Diseases] explode all trees

#5 (cardiovascular disease):ab,ti,kw OR (CVD):ab,ti,kw OR (cardiovascular risk):ab,ti,kw OR (cardiovascular events):ab,ti,kw OR (acute coronary syndromes ):ab,ti,kw OR (ACS):ab,ti,kw OR (coronary artery disease ):ab,ti,kw OR (CAD):ab,ti,kw OR (coronary heart disease ):ab,ti,kw OR (CHD):ab,ti,kw OR (ischemic heart disease):ab,ti,kw OR (ischaemic heart disease):ab,ti,kw OR (myocardial infarction):ab,ti,kw OR (MI):ab,ti,kw OR (myocardial ischemia):ab,ti,kw OR (myocardial ischaemia):ab,ti,kw OR (coronary stenosis):ab,ti,kw OR (coronary restenosis):ab,ti,kw OR (heart failure):ab,ti,kw OR (cerebrovascular disease):ab,ti,kw OR (cerebrovascular disorder):ab,ti,kw OR (stroke):ab,ti,kw OR (cerebral infarction):ab,ti,kw OR (intracranial arteriosclerosis):ab,ti,kw OR (brain ischemia):ab,ti,kw OR (transient ischemic attacks):ab,ti,kw OR (TIA):ab,ti,kw OR (intracranial hemorrhage):ab,ti,kw OR (hemorrhagic stroke):ab,ti,kw OR (death):ab,ti,kw OR (all-cause death):ab,ti,kw OR (mortality):ab,ti,kw OR (all-cause mortality):ab,ti,kw OR (cardiovascular mortality):ab,ti,kw

#6 #4 or #5

#7 MeSH descriptor: [Epidemiologic Studies] explode all trees

#8 MeSH descriptor: [Cohort Studies] explode all trees

#9 (epidemiologic):ab,ti,kw OR (cohort):ab,ti,kw OR (longitudinal):ab,ti,kw OR (follow-up):ab,ti,kw OR (observational):ab,ti,kw OR (prospective):ab,ti,kw

#10 #7 OR # 8 OR #9

#16 #3 and #6 and #10

**Table 1. Listing of endpoints used across included studies.**

| study | clinical endpoints |
| --- | --- |
| Choi 2020 | cardiac death, nonfatal MI, nonfatal stroke, and any revascularization |
| Franco 2020 | time to NSTEACS, STEMI, stroke, or TIA |
| Peng 2020 | hospitalization for UA, coronary revascularization, nonfatal MI, ischemic stroke, and cardiovascular death |
| Zhang 2019 | cardiac death, stroke, recurrent acute MI, and TVR |
| Cao 2019 | MI, stroke, UA, PCI, CABG, peripheral arterial revascularization |
| Gao 2018 | cardiac death, nonfatal AMI, coronary revascularization, and ischemic stroke |
| Cheng 2016 | death and ACS |
| Navarese 2016 | cardiovascular death, MI, UA, stent thrombosis, repeat revascularization and ischemic stroke |
| Li 2015 | the composite of cardiac death, stroke, MI, post discharge revascularization (PCI/CABG), or UA |
| Gencer 2015 | the composite of all-cause death (cardiac, vascular, and non-CV) |
| Werner 2014 | the composite of cardiovascular death and cardiovascular hospitalization for ACS or hospitalization for unplanned, symptom-induced coronary angiography and coronary revascularization (including bypass surgery) |

**Table 2. Listing of adjustments used across included studies.**

| study | adjusted for |
| --- | --- |
| Choi 2020 | age, sex, BMI, hypertension, diabetes, smoking, prior statin use, AMI, LVEF, TG, HDL-C, LDL-C, high-sensitivity CRP, multivessel disease, and culprit lesion |
| Franco 2020 | Not mentioned |
| Peng 2020 | Adjusting for age, sex, BMI, smoking, drinking, hypertension, family history of CAD, Gensini score, TC, LDL-C, HDL-C, TG, FPG, HbA1c, fibrinogen, β-blockers |
| Zhang 2019 | Not mentioned |
| Cao 2019 | age, sex, baseline statin use, smoking, diabetes and hypertension, TC, apoB, lp(a), LDL-C |
| Gao 2018 | mini-GRACE score, sex, current smoking, and diabetes mellitus (the mini-GRACE score accounts for age, SBP, ST-segment deviation, cardiac arrest at admission, elevated cardiac enzymes, and heart rate) |
| Cheng 2016 | age, gender, DM, hypertension, hypercholesterolemia, smoking, clinical presentation (ACS or stable CAD), LDL-C and statin use registered at the time of hospital admission) |
| Navarese 2016 | diabetes, dyslipidemia, age, sex, previous MI, ACEI or ARB at admission and beta-blocker use, type of antiplatelet agent (ticagrelor or prasugrel) |
| Li 2015 | age, sex, BMI, hypertension, diabetes, hypercholesterolemia, hypo HDL cholesterolemia, current smoking, family history of CAD, uric acids, and hs-CRP |
| Gencer 2015 | sex, history of hypertension, history of diabetes, smoking status, baseline total cholesterol and baseline use of statin, GRACE score (ST segment deviation, age, heart rate, SBP, creatinine, Killip class, cardiac arrest at admission and elevated biomarkers of necrosis) |
| Werner 2014 | Age, gender, risk factors, medication, glucose metabolism and lipids |

**Table 3. Listing of enrollment criteria used across included studies.**

| study | clinical endpoints |
| --- | --- |
| Choi 2020 | coronary artery disease patients scheduled for percutaneous coronary intervention |
| Franco 2020 | Acute coronary syndrome patients underwent percutaneous coronary intervention 6 months before |
| Peng 2020 | Patients firstly diagnosed as stable coronary artery disease (based on symptom and coronary angiography and/or coronary computed tomography) |
| Zhang 2019 | acute myocardial infarction patients underwent primary PCI within 24 h of onset |
| Cao 2019 | patients with angiography-proven coronary artery disease |
| Gao 2018 | acute myocardial infarction patients |
| Cheng 2016 | patients underwent diagnostic coronary angiography or percutaneous coronary intervention for acute coronary syndrome or stable angina pectoris |
| Navarese 2016 | Acute coronary syndrome patients |
| Li 2015 | nontreated stable coronary artery disease underwent coronary angiography |
| Gencer 2015 | Acute coronary syndrome patients |
| Werner 2014 | documented clinically stable coronary artery disease patients |

**Table 4. NEWCASTLE - OTTAWA QUALITY ASSESSMENT SCALE**

| study | Selection | | | | Comparability | Exposure | | | total |
| --- | --- | --- | --- | --- | --- | --- | --- | --- | --- |
| Choi 2020 | 1 | 1 | 1 | 1 | 2 | 1 | 1 | 1 | 9 |
| Navarese 2016 | 1 | 1 | 1 | 1 | 2 | 0 | 1 | 1 | 8 |
| Franco 2020 | 1 | 1 | 1 | 1 | 2 | 0 | 1 | 1 | 8 |
| Peng 2020 | 1 | 1 | 1 | 1 | 2 | 1 | 1 | 1 | 9 |
| Cao 2019 | 1 | 1 | 1 | 1 | 2 | 1 | 1 | 1 | 9 |
| Zhang 2019 | 1 | 1 | 1 | 1 | 1 | 1 | 1 | 1 | 8 |
| Gao 2018 | 1 | 1 | 1 | 1 | 2 | 1 | 1 | 1 | 9 |
| Cheng 2016 | 1 | 1 | 1 | 1 | 2 | 0 | 1 | 1 | 8 |
| Li 2015 | 1 | 1 | 1 | 1 | 2 | 0 | 1 | 1 | 8 |
| Gencer 2015 | 1 | 1 | 1 | 1 | 2 | 1 | 1 | 1 | 9 |
| Werner 2014 | 1 | 1 | 1 | 1 | 2 | 1 | 1 | 1 | 9 |

**Figure legends:**

Supplementary Figure 1-2. Subgroup analyses for circulating PCSK9 and the cardiovascular risk.

A: per one standard derivation increase in baseline PCSK9 levels

B: top vs. bottom tertile of baseline PCSK9

Supplementary Figure 3. Sensitivity test of single study on combined effect estimation of cardiovascular events.

Supplementary Figure 4. Funnel plot of the association between circulating PCSK9 concentration and cardiovascular events.
