# Supplementary figures and images for: Association Between Circulating Proprotein Convertase Subtilisin/Kexin Type 9 Concentrations and Cardiovascular Events in Cardiovascular Disease: A Systemic Review and Meta-Analysis
Source: Front Cardiovasc Med. 2021 Nov 23;8:758956. doi: 10.3389/fcvm.2021.758956 (PMC8650021; doi:10.3389/fcvm.2021.758956)

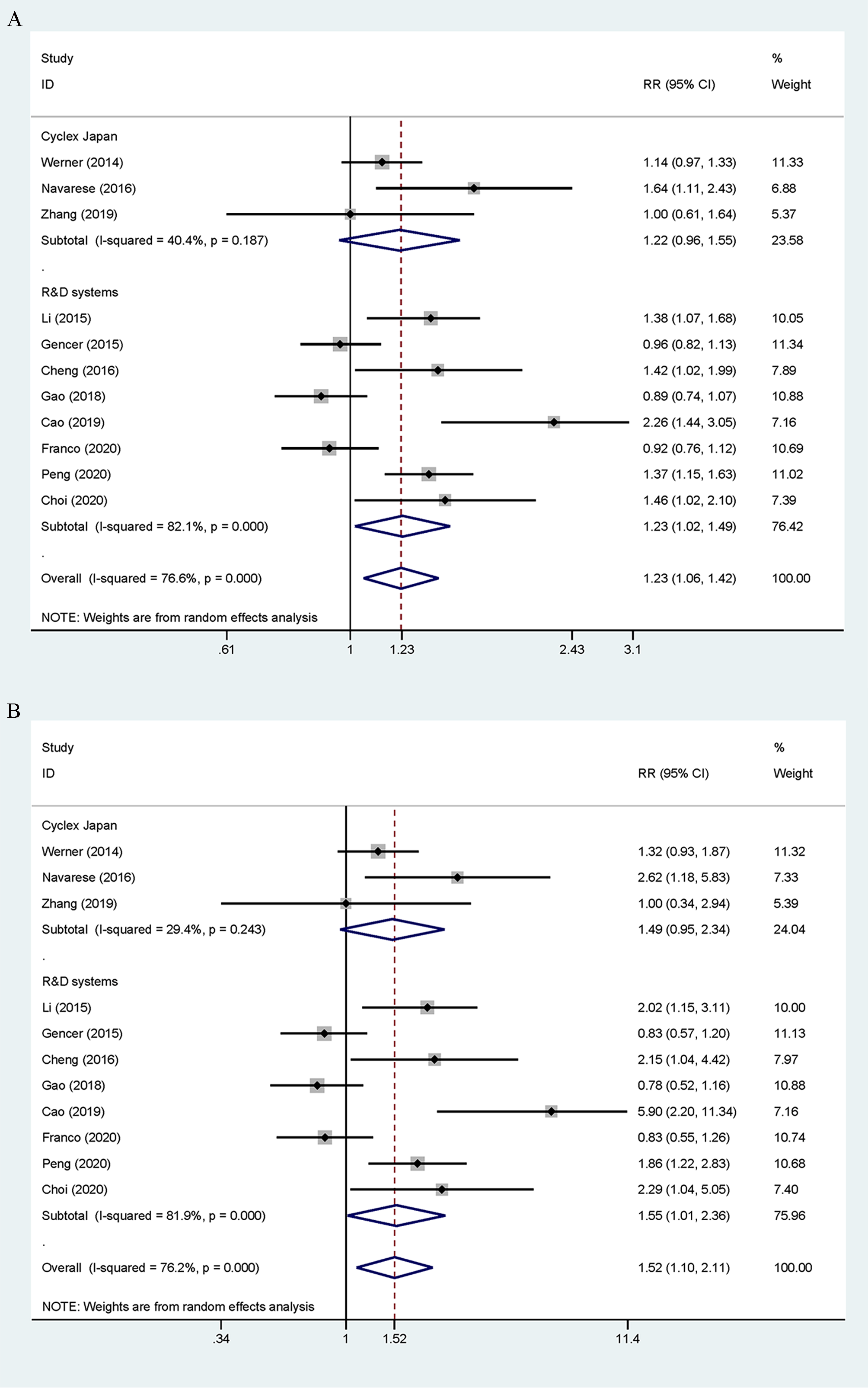

Supplement: Supplementary file 2 [file Image_1.TIF]

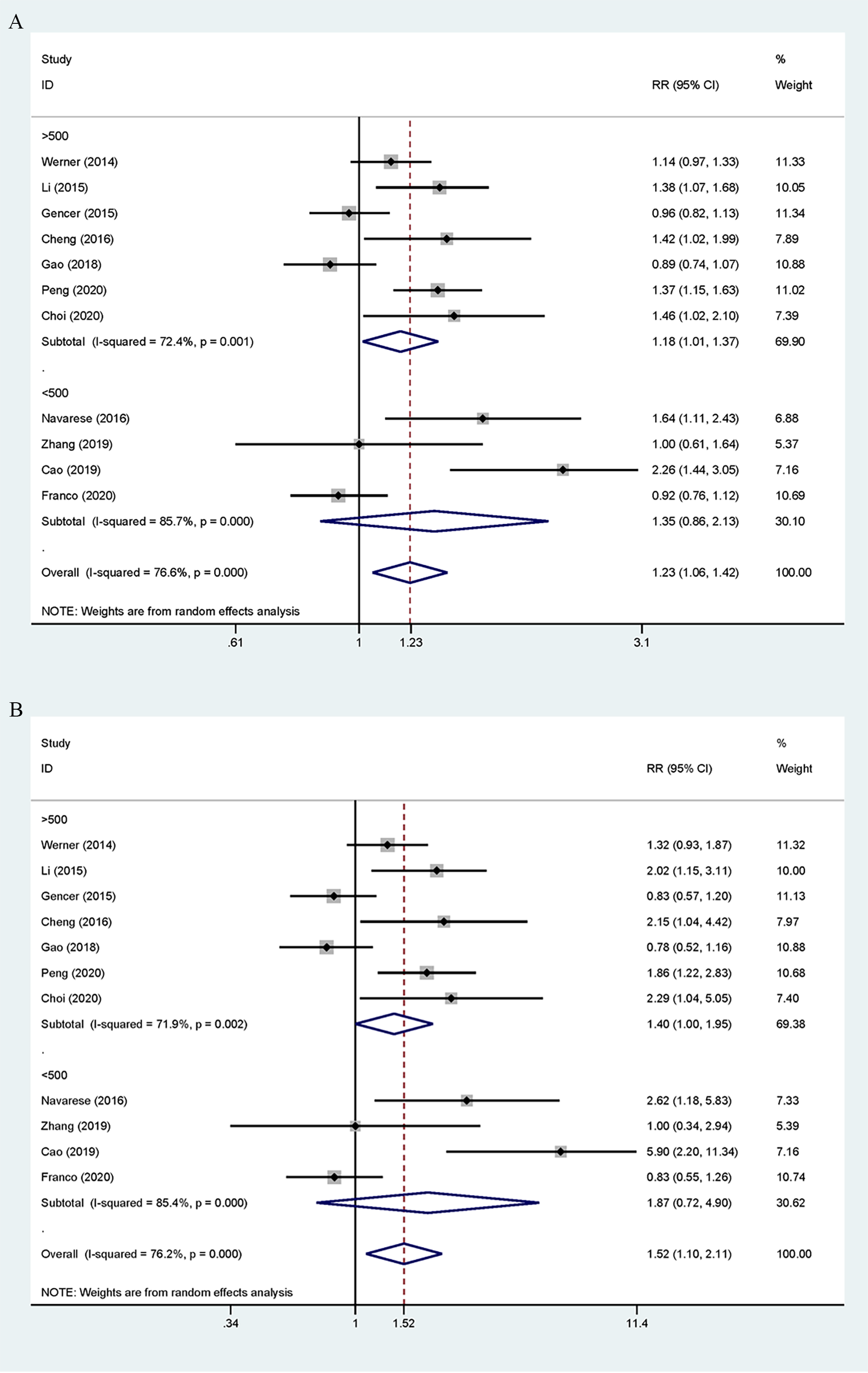

Supplement: Supplementary file 3 [file Image_2.TIF]

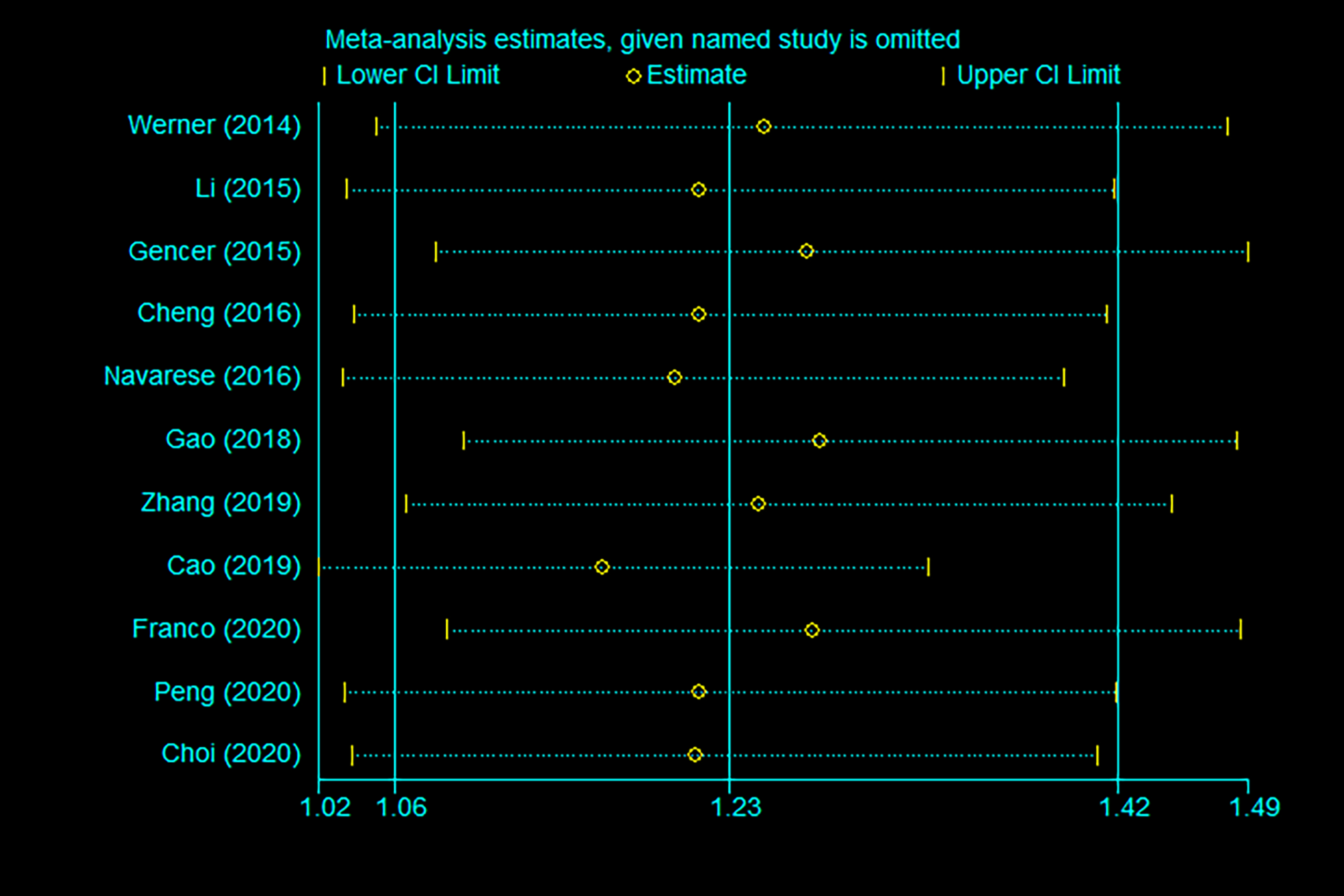

Supplement: Supplementary file 4 [file Image_3.TIF]

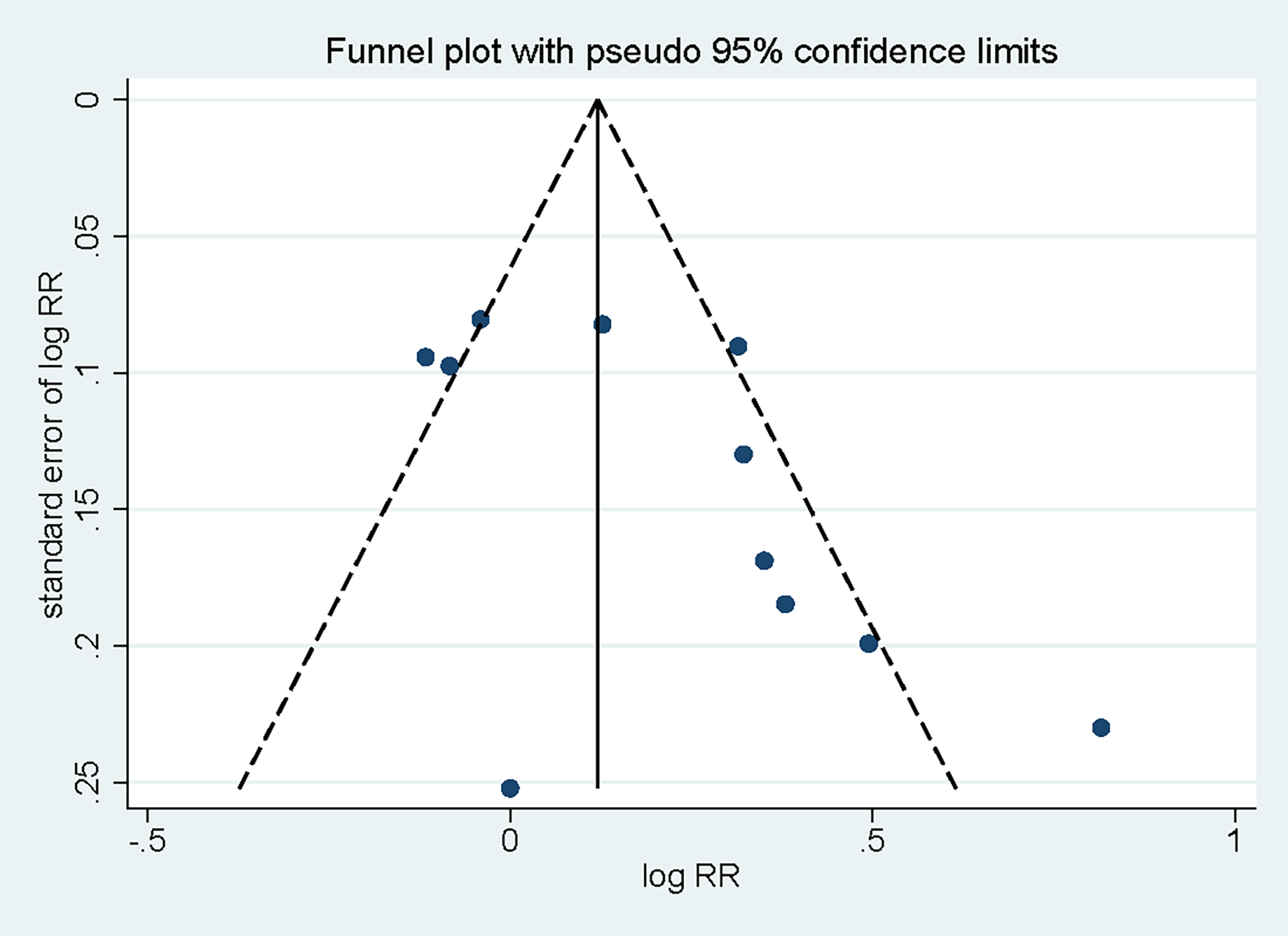

Supplement: Supplementary file 5 [file Image_4.TIFF]
